# History and its relevance to contemporary and future leadership

**DOI:** 10.1136/leader-2024-000993

**Published:** 2024-04-04

**Authors:** Martin Bricknell

**Affiliations:** 1 Centre for Conflict and Health Research, King's College London - Strand Campus, London, UK

**Keywords:** medical leadership, competencies, career development

## Abstract

**Background/Aim:**

This paper argues that an inquisitiveness into the history of medicine and healthcare organisation is an important characteristic of a leader seeking to understand why facts are as they are, before embarking on leading change. I had the privilege of 34 years of service in the UK Defence Medical Services, culminating in the most senior role of Surgeon General. I, and many of my military medical colleagues, are members of the Faculty of Medical Leadership and Management. Through this, I hope that we have been able to add an interesting dimension to the practice of medical leadership in UK health organisations.

**Methods:**

This paper is a reflection on my personal experience suggesting that studying the history of military medicine can provide insights into the collective knowledge of previous generations, the process of organisational development during war, and the clinical and system innovations needed for the next war.

**Results:**

This paper summarises my personal experience of the relevance of the history of military medicine in clinical practice and policy development within the UK Defence Medical Services. It has five sections starting with history as a trajectory of knowledge, and how this links to my personal career. I then show how history informed my leadership influence on policy and practice in four topics: the prevention of heat illness, the organisation of medical services, partnerships in military medicine, and organisational learning. The paper is framed around my personal experience over a career that spanned clinical practice, policy development, leadership on military operations, and finally senior strategic roles.

**Conclusion:**

While I have placed my argument in the context of military medical leadership, I suggest that understanding history is just as important in civilian medical leadership.

## Introduction: history as a trajectory of knowledge

**Figure 1:**
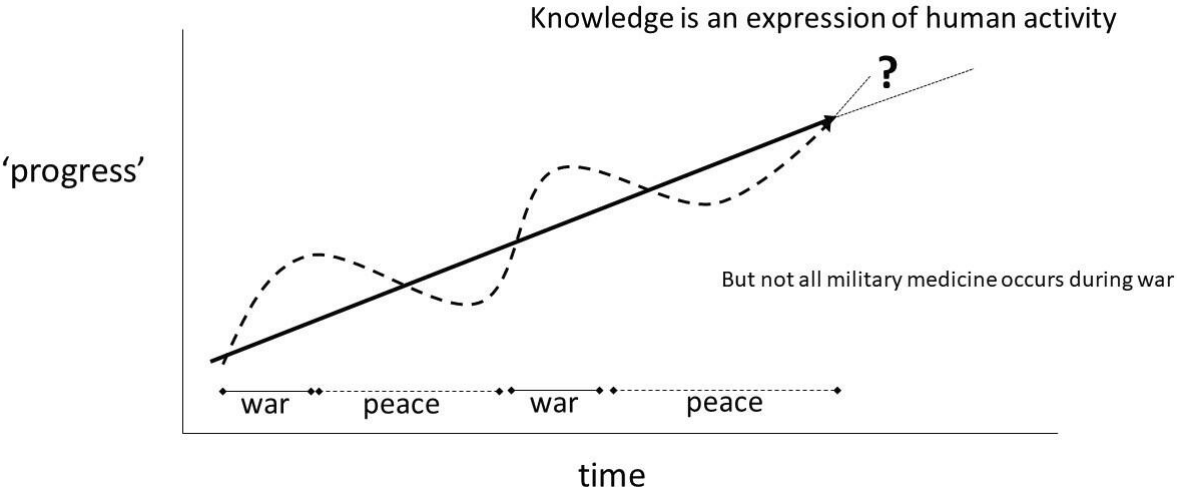
History as a trajectory of knowledge.

 Maintaining clinical and organisational performance in military health systems can be challenging because, fortunately, wars occur infrequently. Medicine is considered by some to be the only beneficiary of war, and, indeed, many advances in clinical medicine have evolved during wartime. I suggest that it is both a duty and an inevitability that the health professions will advance the care of trauma patients during war because of the requirement for a systematised approach to the treatment and evacuation of casualties through many echelons of healthcare. Schematically, this can be considered as a trajectory of medical knowledge as shown in figure 1. Progress takes time to initiate, and an effort to maintain. In peace, which fortunately is the majority of time, military health systems are training for the demands of an ‘epidemic of injuries’ without current clinical experience of the realities of war. This can result in a dip in the clinical performance of a military health system at the beginning of a war compared with that achieved at the end of the previous war because of the limited practical experience of individual healthcare practitioners and the whole system. I suggest that this reduction in performance can be mitigated by careful study of military medical history to interpret the lessons of the past for their relevance to the future. This reflects the same professional duty that non-medical military officers have towards their wider study of the history of war.

The medical history of war, particularly for the 20th century, is replete with sources and resources. The medical volumes of the official history of World War 1 and World War 2 provide a comprehensive review of the clinical and organisational developments of the military medical services. During these wars, many academic papers on the care of casualties from war were published in the *Lancet* and *British Medical Journal*. Beyond these journals, the *Journal of the Royal Army Medical Corps* and the *Journal of the Royal Naval Medical Service* are a contemporaneous record of intellectual thought within the UK military medical services. Historians have also written comprehensive summaries of the medical history of the world wars. This rich legacy should make it easy to access the knowledge from the past to inform the maintenance and generation of new knowledge into the future. I will show how these have been a valuable foundation for my own professional development. David Vassallo and I have recently published a guide to such sources and resources.^1^

So, let me now bring a personal perspective to the relevance of military medical history. I joined the Army in 1987 at the completion of my undergraduate medical degree. My first tour with the Parachute Regiment and subsequent professional training developed my personal skills in prehospital care, primary care, occupational health and public health. For the first decade of the 21st century, I was focused on tactical and operational level leadership with command of a field hospital, chief instructor of the Defence Medical Services Training Centre, and tours in Iraq and Afghanistan leading and managing medical services on military deployments. My final decade of military service in the 2010s concentrated on strategic leadership addressing medical organisational issues, including relationships between military and civilian medical systems within the UK and internationally. I will use topics from each period to illustrate how insights from the past informed my policy and practice for the future.

## Prevention of heat illness

Deaths and significant injuries from heat illness are a particular occupational problem for armed forces because of the need for intense physical training and deployment to hot climates. The issue became personal when I suffered heat exhaustion during training for the Parachute Regiment. Subsequently, while serving in Cyprus as a general practitioner during the early 1990s, we regularly sent heat illness cases to hospital from unacclimatised soldiers undertaking physical activity in the summer. I conducted a small epidemiological study on the incidence of heat illness cases which showed that the majority of cases occurred because commanders were not following the guidance on the prevention of the condition.^2^ Based on this, I conducted a literature review by hand searching Index Medicus for relevant papers supported by an analysis of technical reports, military procedures and policy held at the Army Personnel Research Establishment. This work showed that heat illness has been recognised as a particular military ‘occupational disease’ for over 100 years.^3 4^ The most inciteful finding was that the assessment of risk had not been reviewed since the publication of guidance on the heat stress limits for the prevention of death from heat illness among US Marine Corps trainees in South Carolina in the 1950s. This did not provide heat stress limits for the prevention of hospital admission or collapse from heat illness.

My professional education in occupational medicine provided further opportunities to investigate the topic and address this policy gap. I undertook a series of epidemiological, physiological and ‘real world’ observational studies to propose a new set of pragmatic heat stress limits for the conduct of military activities for acclimatised and non-acclimatised personnel. When I was appointed to the Army Health Unit in 1998, I was able to promulgate a new military policy for the prevention of heat illness as a Defence Council Instruction in 1999 and an update in 2001. While there were very few deaths or serious hospital admissions from heat illness during military operations in Iraq and Afghanistan, there have been several deaths from heatstroke during the UK summers over the last decade. In summary, heat illness among the armed forces will continue to be an enduring occupational risk because of the nature of military training. The control of risk from heat illness is a balance between risk factors, environmental temperature, physical activity. Heat stress limits are an important tool in managing risk but the threshold should be determined by the level of accepted risk. The most recent policy is contained in Joint Service Publication 375, last updated in 2023.^5^ Personally, I learned the importance of understanding the historical background to current policy to determine if policy should change. I also learned the organisational and social processes involved in translating research evidence into policy, and the personal challenge of maintaining enthusiasm for change in the face of institutional inertia over the 6-year period that I was actively studying this topic.

## Organisation of medical services for military operations

My career moved from clinical practice to military medical leadership when I was appointed to command 22 Field Hospital in 1999. At this point, the role of field hospitals in the medical evacuation chain of the Army Medical Services was focussed on the potential evacuation of large numbers of casualties from Germany in the event of a war against Russia. However, the Army Medical Services had actually spent the previous decade deploying field hospitals for overseas operations to the Gulf in 1991, to the Balkans from 1992 and to Rwanda in 1994. I felt that we had lost the institutional insights needed to refresh our thinking about the role of field hospitals in a military casualty evacuation system and that the current organisational structure and equipment for field hospitals was insufficiently flexible to support future military operations.

I had a sabbatical to study the evolution of casualty evacuation in the British Army at the Wellcome Institute for the History of Medicine. This work resulted in a series of papers in the Journal of the Royal Army Medical Corps based on an analysis of the implications of the graphical representation of the casualty evacuation chain in progressive versions of British military medical doctrine. Prior to World War 1, this was a simple linear flow with hospitals as a location for holding casualties with no indication of their clinical role. By the end of World War 1 the system had evolved to show flows for stretcher cases and walking wounded and the use of ambulance cars, railways, barges and ships for the movement of casualties. The clinical role of field hospitals had evolved with Casualty Clearing Stations providing surgery close to the front line and, General and Evacuation Hospitals holding patients until they recovered or preparing them for evacuation to the UK.^6^ Further developments by the end of World War 2 included clinical units such as Field Transfusion Units, Field Surgical Teams and Rehabilitation Centres. This war also emphasised the role of the ‘control posts’ for the regulation and distribution of casualties.^7^ Unfortunately, these sophistications from the World War 2 experience had been lost over the subsequent 50 years.

Over first decade of the 21st century, I was able to influence transformation both in the Army field hospital and in wider clinical care across the casualty evacuation chain. Through experimentation, my unit demonstrated new approaches to the deployment and organisation of Army field hospitals. Building on further experience in Iraq and Afghanistan, Army Medical Services doctrine shifted from the transport problem of large numbers of patients to the clinical problem of treating patients with very severe traumatic injuries. The field hospital became the focus of clinical innovation with hospital care being shifted forward from the facility into the ‘prehospital’ helicopter through the creation of the Medical Emergency Response Team. Hospital care also shifted backward with the development of the Critical Care Air Support Team, providing intensive care support inside aeroplanes. Organisationally, we also re-discovered the importance of ‘control posts’ by creating the Patient Evacuation Co-ordination Centre. This recovery of institutional knowledge was reflected in academic papers, revised military medical doctrine, and a new ‘casualty evacuation chain’ titled the ‘Operational Patient Care Pathway’ that reflected clinical requirements rather than organisational structures.^8^ It was also the topic of my PhD. These changes were embedded into new organisational structures during the fundamental redesign of the whole Army as part of the 2010 Defence Review.

This topic of the evolution of the organisation of medical services is probably the central theme in military medical history. I learned that the organisational structures of the military medical services should be driven by the design of clinical teams that provide each element of the care pathway for sick and injured soldiers. The structure must be sufficiently flexible to allow tailoring for specific military missions. The opportunity to fundamentally change structures only occurs in the context of Defence Reviews. Therefore, it is necessary to anticipate the political cycle and have the plan for change ready for the next opportunity.

## Partnerships in military medicine

Although my deployments to Afghanistan in 2006 and 2010 re-emphasised the importance of local civilian and military partnerships during war, I had already become interested in the topic through my deployment to the Balkans in 1996. The wars in Iraq and Afghanistan re-framed this issue in the context of counter-insurgency operations as part of a ‘hearts and minds’ campaign that links security, development, and peace into a strategy. This is not new, and international military medical services made important contributions to the development of local military and civilian health services during and after previous wars of the 20th century. Regeneration of national public health services was an important element of post-conflict recovery in Western Europe after World War 2, and a particular responsibility of the ‘occupying powers’ in West Germany until the restoration of sovereign government. The history of the Korean War and Vietnam War also includes the role of USA and wider international partners in supporting local health services to meet the needs of the civilian population during wartime. History tells us that civil–military and military–military partnerships are a vital element of military healthcare operations.

I was able to use the experience from the conflict in Afghanistan to justify civil-to-military and military-to-military partnerships as a recognised diplomatic task for the UK armed forces within the concept of Defence Health Engagement.^9^ The British military contribution to the international response to the Ebola crisis in 2014 was characterised by relationships with international civilian non-government organisations such as Save the Children, and local Sierra Leonean military and civilian health services.^10^ We extended our thinking in this field as part of the UK deployment of a field hospital to South Sudan within Operation TRENTON from 2017 to 2018. Our goal was to set the conditions for the UK to handover this responsibility to Vietnam. We ran a partnership programme with the Vietnamese armed forces alongside the deployment to South Sudan and handed over the hospital in October 2018.^11^

In reality, military medical services have always had an interest in the indigenous health situation. Indeed the history of the Army Medical Services in the 17th, 18th and 19th centuries is all about endemic infectious diseases and their impact on the health of expatriate British military forces and civilians. We have always fought wars with partners and allies, and have had to consider the medical arrangements for their casualties alongside our own. I believe that both historical and contemporary evidence shows that supporting the development and reconstruction of indigenous health services is a key ‘post-conflict’ activity and one that the military medical services need to train for by education in global health and health systems. Although I knew the UK NHS model is an isolated outlier in global health systems design, I learned that in many countries access to healthcare can be a commodity of political power. We need to be humble in our approach to ‘health systems strengthening’ with international partners and to understand that our own historical journey towards citizens’ universal access to healthcare is not the global norm.

## Organisational learning

My final topic is organisational learning. While this is linked to the previous topic, I consider it to be much broader in order to address the issue of ‘forgetting’ as well as ‘learning’ lessons from experience. I suggest that evidence of organisational learning can be found in the historical artefacts that exist within an institution. This can be the history and doctrine documents that I discussed earlier, but might also include other documents such as textbooks, reports, standardised procedures or academic papers. These artefacts are of critical importance for students of military medical history. Thus, it is an obligation of the contemporary generation to record their experience and learning for the next generation. It is only by ‘joining these dots’ over time that trendline for the development of knowledge from figure 1 can be plotted. It is also important to consider how these artefacts are kept and safeguarded for the future. Past generations produced physical documents which are now available through formal archives and personal collections. As we adopted computers and electronic communication, the modern digital artefact is much more difficult to identify and retain. In my experience, the digital footprint for the development of knowledge in military medicine is likely to be more robust from publishing in the academic literature than government publishing. This almost brings us into a full circle in which I previously noted the importance of translating academic research evidence into actual formal policy, but now the identification of the existence of such policy might only occur through citations in the academic literature because of the constraints on the availability of information from the Ministry of Defence.

**Figure 2:**
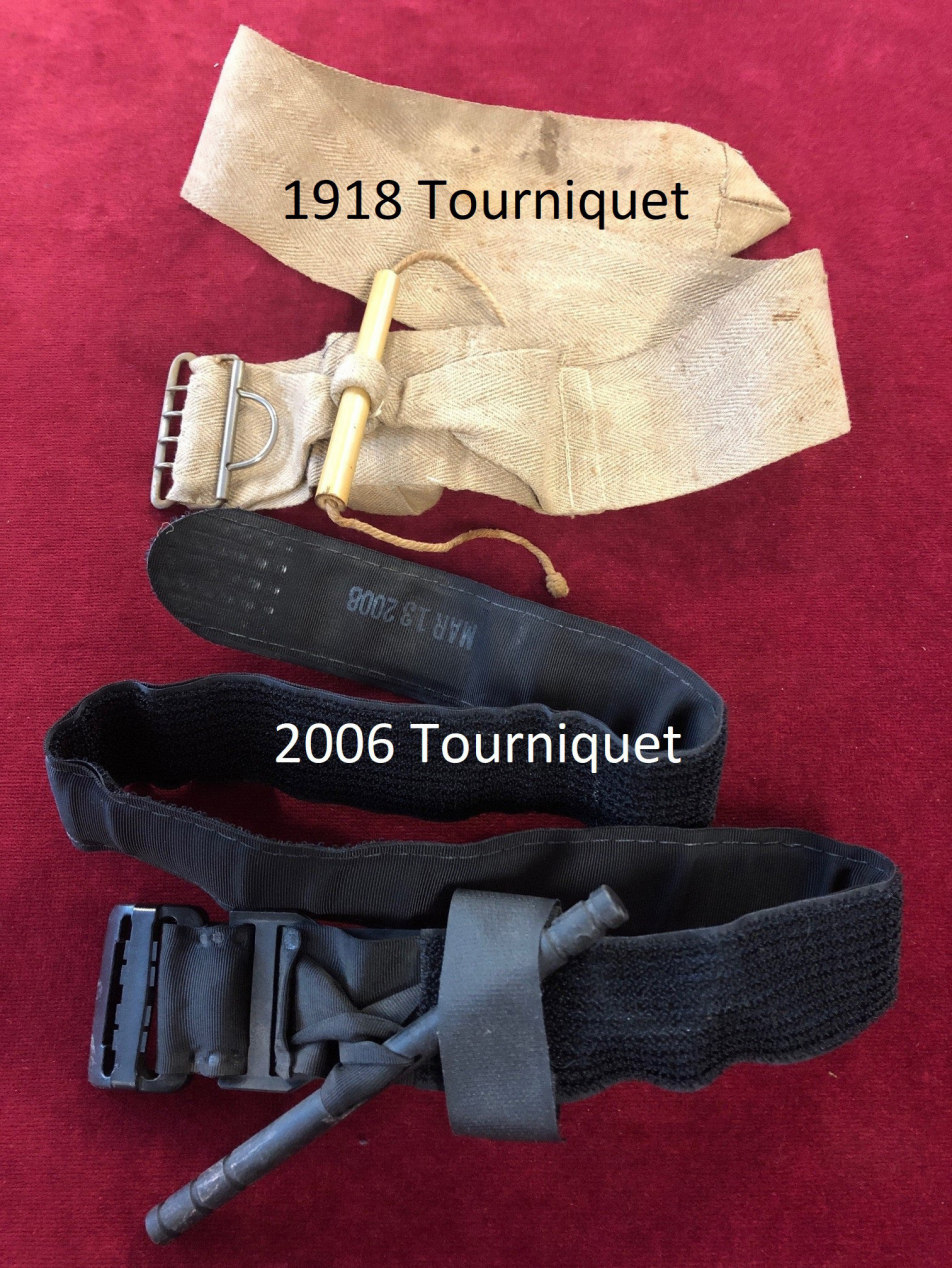
A 1918 and a 2006 military tourniquet.

By taking the highest-level perspective of organisational learning as our frame of reference for military medical history, we can see that medical research and innovation is a key function within military health systems. This is not only important during war, but the occupational environment of military service and the integration of a military health system provides unique opportunities for research in peace. However, converting knowledge into learning requires more than research, this knowledge needs to be converted into policy and training. Through the analysis of the evolution of knowledge we can observe how the relationship between the health of the armed forces, its medical services, and the executive leadership has many continuities. This includes the need for military medical services to record, interpret, and present data on the well-being of armed forces personnel to influence the policies on the wider determinants of health including food, housing, physical training and social culture. It also allows us to identify when lessons have been forgotten, such as the need to reintroduce the same design for a military tourniquet in 2006 as had been issued in 1918—the only difference being the use of Velcro.^12^ The two tourniquets are shown in figure 2.

## Conclusions

I hope that I have shown how study of military medical history during my career allowed me to place contemporary challenges into the continuous trajectory of knowledge in military medicine. I suggest that an inquisitiveness into the history of medicine and healthcare organisation is an important characteristic of a leader seeking to understand why facts are as they are, before embarking on leading change. Reflecting back on my experiences during my career, I can see that organisational learning occurs through the activities of individual people responding to their contemporary environment. The extent to which they divine new information or just re-discover old knowledge is determined by their understanding of their place in this trajectory through the study of military medical history. I suggest that the study of military medical history is a valuable activity for military health professionals. It can help to place current issues in the context of a longitudinal perspective. It can inform current practitioners on the nature of enduring problems alongside changes in the character of a problem. I also believe that it is a duty of those in the present to record their experience and knowledge through academic publication so maintaining the continuity of the historical record for the next generation by providing a linking trajectory from the past through to the future. My primary regret is that I was not able to formally mandate the study of military medical history as part of the professional training of military medical services personnel. While I have placed my argument in the context of military medical leadership, I suggest that it is just as important in civilian medical leadership. Unfortunately, medical history does not currently feature in contemporary health professional education.

## Data Availability

No data are available.
